# Identifying anti-TNF response biomarkers in ulcerative colitis using a diffusion-based signalling model

**DOI:** 10.1093/bioadv/vbab017

**Published:** 2021-08-18

**Authors:** Amrinder Singh, Endre Anderssen, Christopher G Fenton, Ruth H Paulssen

**Affiliations:** 1 Clinical Bioinformatics Research Group, Department of Clinical Medicine, UiT-The Arctic University of Norway, Tromsø N-9037, Norway; 2 Genomics Support Centre Tromsø (GSCT), Department of Clinical Medicine, UiT-The Arctic University of Norway, Tromsø N-9037, Norway

## Abstract

**Motivation:**

Resistance to anti-TNF therapy in subgroups of ulcerative colitis (UC) patients is a major challenge and incurs significant treatment costs. Identification of patients at risk of nonresponse to anti-TNF is of major clinical importance. To date, no quantitative computational framework exists to develop a complex biomarker for the prognosis of UC treatment. Modelling patient-wise receptor to transcription factor (TF) network connectivity may enable personalized treatment.

**Results:**

We present an approach for quantitative diffusion analysis between receptors and TFs using gene expression data. Key TFs were identified using pandaR. Network connectivities between immune-specific receptor-TF pairs were quantified using network diffusion in UC patients and controls. The patient-specific network could be considered a complex biomarker that separates anti-TNF treatment-resistant and responder patients both in the gene expression dataset used for model development and separate independent test datasets. The model was further validated in rheumatoid arthritis where it successfully discriminated resistant and responder patients to tocilizumab treatment. Our model may contribute to prognostic biomarkers that may identify treatment-resistant and responder subpopulations of UC patients.

**Availability and implementation:**

Software is available at https://github.com/Amy3100/receptor2tfDiffusion.

**Supplementary information:**

Supplementary data are available at *Bioinformatics Advances* online.

## 1 Introduction

### 1.1 About disease

This paper focuses on ulcerative colitis (UC) a subtype of inflammatory bowel disease (IBD) along with Crohn’s disease. UC is a complex chronic inflammatory disease with dysregulation of the immune responses in the colonic mucosa. The disease features chronic acute relapsing disease activity, with intervals of remission (Khor *et al.*, 2011). Emerging evidence implicates immunological, microbial, environmental and genetic factors in the disease pathogenesis (Zhang and Li, 2014). Analysis of UC risk genes from genome-wide association studies (GWAS) implicates processes such as cell–cell communication, response to cytokine stimulus, and cell surface receptor intracellular signalling (Jostins *et al.*, 2012). Targeted treatments that induce remission in subpopulations of UC patients act by inhibiting signalling pathways between extracellular signalling molecules such as cytokines, and key transcriptional regulators of inflammatory processes (Schwartz *et al.*, 2017). However, there is significant patient-to-patient variability in treatment response, as shown by the low response rates in clinical trials (Hindryckx *et al.*, 2015; Jairath *et al.*, 2015). Therefore, we seek a method of quantifying patient-specific differences through receptor to transcription factor (TF) signalling.

### 1.2 Disease biomarkers

Successful personalized medicine for UC requires accurate biomarkers that can identify resistant and responders, but no individual molecular biomarker is currently recommended for clinical use to predict the treatment effects in UC (Kim *et al.*, 2017). Patient-specific biomarker discovery methods are prone to overfitting, resulting in the identification of clinically unreliable biomarkers (Hernández *et al.*, 2014). Embedding biological information from networks in the biomarker discovery process may reduce the risk of overfitting (Guo and Wan, 2014).

### 1.3 Proposed method

**Fig. 1.**
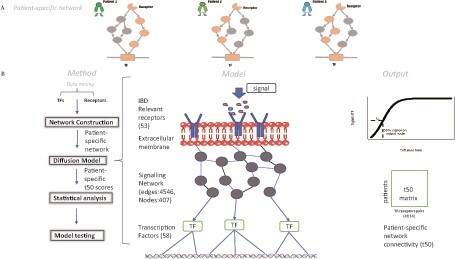
Outline of diffusion model. (**A**) A schematic figure illustrating how the same biological pathway associated with a specific function may be perturbated leading to a different route of signal transduction from receptor to TF in different patients. This model can be adapted to data to build a patient-specific model (alternatively, the model can be completely generated from the data). The model can then be used to generate predictions of therapies for the patient. (**B**) Concept of diffusion model based on calculating patient-specific network edge weights in a network connecting cytokine receptors to TFs through a protein-PPI network. In the diffusion process, receptors receive a signal, and it diffuses through the network to the TFs. The model output is signal received by the TFs over time which is simplified as time to reach 50% of maximum signal at the TF (*t*_50_). The output *t*_50_ data matrix contains *t*_50_ values for each receptors-TF pair per patient. This matrix can then be used for further statistical analysis or machine learning

In this study, we propose quantifying patient-specific network connectivities between pairs of genes as complex biomarkers. However, with over 20 000 genes in the human genome, the number of potentials pairwise connections approaches 200 million. Therefore, it is necessary to identify a limited number of biologically relevant connections that explains a plausible biological mechanism central to UC aetiopathogenesis. This prevents overfitting caused by the large number of potential connections. Hence, we focus on the network connectivities between disease-relevant receptors and TFs that regulate the expression of genes involved in the inflammatory process (Fig. 1A and B). This connectivity can be quantified by network diffusion. Network diffusion describes the gradual spread of an abstract signal throughout a network. Diffusion is a global network process that considers all available paths, not just direct links or the shortest paths (Di Nanni *et al.*, 2020). Thus, the diffusion time represents the overall network connectivity between two genes, e.g. from a receptor to a TF (Fig. 1A).

## 2 Methods

The methods are briefly described (see the Supplementary Method for details). Statistical analysis and processing of the data were performed using R version 3.6.3 (www.r-project.org). To identify relevant TFs, the Bioconductor R package, pandaR (10.18129/B9.bioc.pandaR; Schlauch *et al.*, 2017) was used. IBD-relevant cytokines were selected from the list of GWAS risk genes for IBD (Supplementary Table S1). The comPPI database (Veres *et al.*, 2015) was used to create a signalling network connecting receptors to TFs. Network diffusion was performed on this network to estimate network connectivity between each receptor-TF pair. The differential connectivity between sample groups was tested using linear modelling (Ritchie *et al.*, 2015).

### 2.1 Initial data mining

The Gene expression Omnibus (GEO) was searched for datasets containing gene expression data from the colon biopsies obtained before treatment with anti-TNF and with treatment response data available. The detailed search protocol is available in the Supplementary Methods. Gene expression data for mucosal gene expression in IBD before and after treatment with anti-TNF (infliximab) were downloaded from the GEO (Supplementary Table S2). GSE16879 was used as a training dataset for model development (Arijs *et al.*, 2009a). The remaining datasets were used for testing.

Regulatory motif binding information was obtained from the regulatory circuits database (Marbach *et al.*, 2016), which contains available TF binding sites in several tissues and cell types. The binding motif-set representing general immune cells (high-level network ‘14_immune_organs.txt’) was chosen from a regulatory circuits database as a relevant representation of the inflammatory cells involved in UC. Protein–protein interaction (PPI) data were obtained from the ComPPI database (Veres *et al.*, 2015). This is a cellular compartment-specific database of proteins and their interactions (http://ComPPI.LinkGroup.hu). Only interactions with a confidence score >0.6 were used in the network construction.

To identify key TFs, pandaR (Passing Attributes between Networks for Data Assimilation), was applied to the training Gene expression dataset (Schlauch *et al.*, 2017). pandaR creates a gene regulatory network (GRN) with weighted edges between TFs and gene targets regulated by these TFs. To evaluate which TFs significantly contributed to the variation in gene expression, a null distribution regulation network edge weight was computed by randomizing the TF gene target information. Then, the resulting null distribution was used to calculate an empirical *P*-value for each TF.

A sub-network was extracted from the PPI connecting key TFs to cytokine receptors. Genes annotated with the transducer Gene Ontology (GO) terms: GO:0002768 (cell surface receptor signalling pathway) or GO:0019221 (cytokine-mediated signalling pathway) were included in the intermediate network between the TFs and receptors. In the resulting signalling network, the nodes represent the genes coding for the interacting proteins, and the edges represent physical interactions that may pass a biological signal. ComPPI was used to obtain PPIs. The network includes interactions involving the selected TFs, surface receptors which are known UC risk genes and signal transduction genes, such as kinases, that may contribute to passing information between the receptors and TFs. The same signalling network was used for all the UC datasets.

### 2.2 Reference methods for biomarker discovery

To separate treatment-resistant and responder patients, we initially tried the biomarker discovery tool LIONESS which quantifies patient-specific GRNs (Kuijjer *et al.*, 2019) and ‘nnet’ a deep learning-based method (Venables, 2002). The neural network parameters were optimized over a grid of the number of hidden nodes (size) and regularization (decay) parameters. We used ‘nnet’ with 10-fold cross-validation repeated 20 times using average accuracy to select the final model. This process was repeated across a grid of number of hidden nodes and the regularization parameter to identify the optimum model structure for each dataset. Prediction results in the testing datasets were evaluated by area under the receiver operating curve (AUC). In the algorithm, other parameters were kept at their default settings.

### 2.3 The diffusion model

We chose to model the results of the biochemical events that occur during signal transduction using a network connecting cell surface receptors to TFs in the nucleus. The model is adapted from Fick’s law of chemical diffusion to a network structure. See e.g. Philibert (2005) for a review. Consider a patient-specific signalling network with nodes representing proteins e.g. cytokines, receptors and kinases create a signal transduction cascade. If a signal S, analogous to a concentration of a chemical in Fick’s law, is placed on a node i, the signal flux *F* along a network edge connecting node i to node j at a time t is given by:

$${F\left(t\right)}_{i\to j}\;={(S}_{i}-S_{j})\times E_{i}\times E_{j}.$$

Where the edge connectivity weight, analogous to the diffusion constant in Fick’s law, is calculated using the patient’s normalized gene expression values, E, of the genes coding for the proteins i and j. The signal present at each protein node i connected to *J* other protein nodes j ϵ 1.J is then updated at time t + 1 using the sum of all fluxes:

$${S\left(t+1\right)}_{i}={S\left(t\right)}_{i}\;+\sum_{j=1}^{J}{F\left(t\right)}_{i\to j}.$$

The computation is initialized by setting all signal levels to zero and then placing one unit of signal on a starting receptor protein. The signal propagates through interconnected proteins throughout the network. To quantify the connectivity, we take the number of time steps to reach 50% of the maximum signal at the TF of interest ${(t}_{50})$. This methodology was implemented in R (4.1.0). Simulations were run for 2000 timesteps for all samples in each dataset, generating a new data matrix of $t_{50}$ data with rows for each sample and a column for each receptor-TF pair. To evaluate if the obtained matrix of diffusion data contains new information or is merely a linear combination of the original gene expression data, the $t_{50}$–feature space was compared to the original gene expression matrix using Procrustes rotation (Peres-Neto and Jackson, 2001). This method was also used to test if the $t_{50}$ data reflected the gene expression levels of just a few highly connected or ‘hub’ genes or global gene expression changes due to variations in proliferation rate or infiltration of immune cells. Cell deconvolution was used to estimate the infiltration of different immune cells in all samples (Becht *et al.*, 2016) and a gene expression signature (Sotiriou and Pusztai, 2009) was used to estimate the proliferation rate.

### 2.4 Statistical analysis

Significance testing for differentially expressed genes, regulatory network connectivities and diffusion ${(t}_{50})$ on the training dataset was performed using limma (Ritchie *et al.*, 2015).

Patients were grouped as normal controls, i.e. non-UC diagnosis. Responders, which attained a complete mucosal healing with a decrease of the Mayo endoscopic subscore and histological score to 0 or 1. Patients that did not attain the mentioned level of response were placed in the resistant group despite some of them showing endoscopic or histologic improvements (Arijs *et al.*, 2009b).

Three comparison tests were made:

1. Inflamed versus non-inflamed: To identify pathways that may be involved in active inflammation, we compared samples from patients with active endoscopic inflammation to non-UC controls and UC patients that had responded to treatment. The inflamed group comprised all patient samples taken from an active site of inflammation before treatment or from a treatment-resistant patient after treatment. The non-inflamed comprise normal control samples (*N* = 6) and responders after treatment (*N* = 8).
2. Resistant versus responder: To look for a biomarker of drug response, we compared samples from resistant and responder patients obtained before treatment.
3. Male versus female. As a negative control of samples concordant for inflammation, we compared samples obtained from males and female patients before treatment.

Correcting for multiple testing was done with the method of Benjamini and Hochberg (1995). Exploratory data visualization was done using principal component analysis (PCA) and Partial least squares (PLS) regression (Gidskehaug *et al.*, 2007). Gene annotation was performed using the Bioconductor org. Hs.eg.db package version 3.12.0 [10.18129/B9.bioc.org.Hs.eg.db]. GO enrichment analysis was performed using the clusterProfiler, Bioconductor package (Yu *et al.*, 2012).

## 3 Results

### 3.1 Data mining and network definition

Fifty-three IBD-relevant cytokines were selected from the 1067 identified GWAS risk genes for IBD (Supplementary Table S3). Key 58 TFs were identified using the sum of their regulatory network connections from pandaR (empirical *P*-value < 0.05). A list of the selected TFs and receptors is available in the supplements (Supplementary Table S3), and a full list of all TFs considered with their annotations and relevant target genes (Supplementary Tables S4 and S5). The comPPI database (Veres *et al.*, 2015) was used to create a signalling network connecting the cytokines to the TFs through 83 receptors and 266 intracellular signal transduction proteins generating a signalling network with 407 nodes and 4546 edges (Fig. 1B).

### 3.2 Diffusion model creates a feature space that contains novel information compared to gene expression

The diffusion model describes network connectivity from receptors to key TFs, using the time it takes a signal to diffuse from the receptor to the TF (Supplementary Fig. S1 for an example), generating a new feature space of 4814 receptor-TF pairs. Procrustes rotation was used to compare this feature space to the original gene expression space and estimate the fraction of $t_{50\;}$ variability that is linearly dependent on gene expression (Table 1). Overall, in the four datasets examined, between 70% and 80% of the $t_{50}$ information is directly linearly dependent on the gene expression data. To investigate if the $t_{50}$ data were primarily driven by highly connected ‘hub’ genes in the signalling network, we extracted a subset of gene expression data for 46 genes with more than 50 edges. These hub genes could explain between 60% and 75% the variability in the $t_{50}$ data. In comparison, the hub genes could explain between 71% and 87% of the total gene expression data variability.

**Table 1.** Dataset comparisons using Procrustes rotation

Dataset	GEO Acc#	Expression (%)^a^	Hub genes (%)^b^	Global (%)^c^	Expression and hub genes (%)^d^
Training	GSE16879	79	75	64	87
Test	GSE12251	79	75	64	87
Test	GSE23597	71	66	47	71
Test	GSE73661	69	60	48	77

*Notes*: Percentage of $t_{50}$ dataset variability linearly explainable by (a) gene expression of all genes, (b) expression of genes that are highly connected in the signalling network (hub genes with more than 50 edges), (c) global expression changes due to changes in proliferation rate or immune cell infiltration. For comparison, (d) Percentage of gene expression data not explainable by the highly connected hub genes.

Global changes in gene expression can be caused by large-scale tissue changes such as immune cell infiltration or changes in the proliferation rate. To investigate if this controlled the $t_{50}$ data, we estimated the proliferation rate using a proliferation gene expression signature (Hamed *et al.*, 2015) and immune cell infiltration using a cell deconvolution tool developed for tissues analysed using Affymetrix data (Becht *et al.*, 2016). These data were compared to the $t_{50}$ data in the same manner as the gene expression data, but it could explain only from 57% to 64% of the diffusion data (Table 1).

### 3.3 Diffusion model outperforms LIONESS and ‘nnet’ for predicting anti-TNF response in UC

**Fig. 2.**
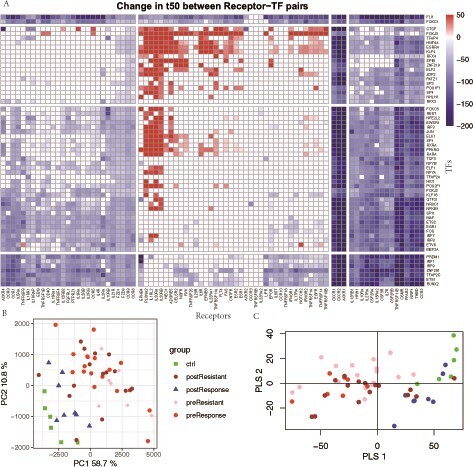
Diffusion time ($t_{50}$) change between receptor-TF pairs in active UC and normal or responders after treatment. (**A**) Heatmaps shows the change in diffusion time in each pair of receptors (*x*-axis) and TFs (*y*-axis) between UC and normal or responders after treatment. Blue shows faster diffusion i.e. better signalling and red shows increased diffusion time i.e. a weakening strength of the connection between the receptor and the TF. (**B**) PCA of diffusion time (*t*_50_) of all receptor–TF pairs and all samples in the training dataset. Normal controls and postresponse samples cluster together in contrast to the active UC samples. (**C**) PLS of the same dataset shows improved separation between preresponse and postresistant groups

We used a linear model to relate $t_{50}$ to inflammation status and drug response in the training dataset. This enabled us to identify the receptor-TF pairs significantly related to active UC. We obtained 2362 receptor-TF pairs with adjusted *P*-values less than 0.01 (Fig. 2A).

**Fig. 3.**
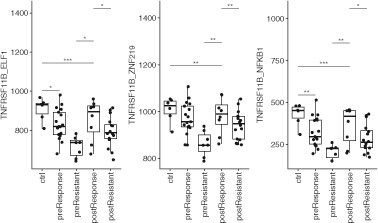
Box plots of $t_{50}$ data for the different patient groups. This figure indicates receptor–TF pairs with top AUC score in UC training dataset

PCA of network connectivities ${(t}_{50}$) shows postresponders clustered with the control group as expected but shows no clear separation between resistant and responders before treatment (Fig. 2B). Using PLS of $t_{50}$, we obtained some separation of treatment-resistant and responder patients (Fig. 2C). We used a linear model to relate the $t_{50}$ data to the anti-TNF treatment-resistant and responder patient groups and identified 114 receptor-TF pairs with significant differences in network connectivity (adj. *P*.val < 0.05; Supplementary Table S6). Using the receiver operating characteristic curve (ROC), we evaluated the individual receptor-TF pairs for their ability to discriminate anti-TNF resistant from responders. We found 35 receptor-TF pairs with AUC higher than 0.84 in the training dataset (Supplementary Table S7). The top-scoring discriminators in the training UC dataset were the receptor-TF pairs TNFRSF11B-ELF1, TNFRSF11B-ZNF219 and TNFRSF11B-NFKB1, each with an AUC of = 0.91 (Supplementary Fig. S2). These pairs show distinct differences between treatment resistant and responders and between resistant and controls (Fig. 3, Supplementary Fig. S2 and Table 2). As a negative example, we compared male (*n* = 14) and female (*n* = 10) patient samples before treatment and found no significant differences in network connectivity (adj. *P* < 0.05). We then tested the ability of the top three receptor-TF pairs to predict anti-TNF response in the test datasets (Supplementary Table S2). The predictive ability of these receptor-TF pairs was compared with a deep learning method, ‘nnet’, a feed-forward neural network algorithm trained on the same dataset. We also tested LIONESS, a method for computing sample-specific GRNs. Surprisingly, the diffusion model outperformed the neural network, giving higher AUC scores (Table 2) in the UC training dataset and the majority of the independent test datasets. We compared inflamed versus noninflamed, i.e. normal controls and responders after treatment versus before treatment and resistant after treatment, in addition to the treatment resistant versus responders. LIONESS estimates a total of 2 678 095 regulatory edge weights per sample. Significantly changed edge weights were then identified using linear modelling (limma). Between the inflamed and noninflamed samples in the training set, 161 052 statistically significant edge weights (adj. *P*-value < 0.01) were found. However, no significant results were obtained (adj. *P*-value ∼ 0.99) for the more important comparison of anti-TNF resistant versus responder comparison (Supplementary Table S8). LIONESS was therefore not applied to the test datasets.

**Table 2.** Testing of predictive ability of diffusion model compared to neural network modelling in Training and Test datasets

Dataset	GEO Acc#	nnet-AUC	TNFRSF11B-ELF1	TNFRSF11B-NFKB1
Training	GSE16879	0.80	0.91	0.91
Test	GSE12251	0.77	0.88	0.78
Test	GSE23597	0.72	0.66	0.59
Test	GSE73661	0.50	0.65	0.68

*Notes*: nnet-AUC shows AUC scores calculated by ‘nnet’. Columns represent receptor-TF pairs TNFRSF11B-ELF, TNFRSF11B-NFKB1, TNFRSF11B-ZNF219 having the best AUC scores using diffusion model on the training dataset.

### 3.4 Validation with rheumatoid arthritis

To assess our model’s generalizability for other autoimmune diseases, we applied the diffusion model to the rheumatoid arthritis (RA) dataset. UC and RA share many inheritable risk loci and have many overlapping pathogenic pathways (Bae *et al.*, 2017; Halling *et al.*, 2017; Hemminki *et al.*, 2009). Therefore, we selected and merged two RA gene expression datasets: GSE24742 (Ducreux *et al.*, 2014) and GSE45867 (Gutierrez-Roelens *et al.*, 2011) to validate our modelling method. The chosen dataset is a microarray gene expression study of paired synovial biopsy samples collected before therapy (T0) and after therapy (T12) from the affected knee of RA patients treated with tocilizumab (TCZ), methotrexate (MTX) or rituximab (RTX). The experiment design of the validation dataset was similar to the UC dataset in terms of before treatment biopsy, underlying disease mechanism (inflammation), and a sufficient number of samples used in the study. We used a dataset with 86 RA samples to validate the model for testing the performance of the model.

**Fig. 4.**
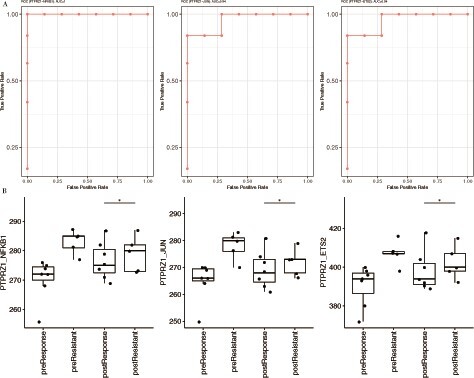
Validation with RA dataset. ROC analysis shows the separation of treatment responder and resistant sample groups. It is calculated based on the *t*_50_ score of receptor–TF pairs (**A**) PTPRZ1-NFKB1 (**B**) PTPRZ1-JUN (**C**) PTPRZ1-ETS2 with the top AUC scores of 1, 0.94 and 0.94, respectively. Box plot illustrates receptor–TF pairs with top AUC score (**D**) PTPRZ1-NFKB1 (**E**) PTPRZ1-JUN (**F**) PTPRZ1-ETS2 which show distinction between treatment response groups based on *t*_50_ score calculated by diffusion model

For validation, we used the same pipeline developed with the same score thresholds. We created a literature curated list of RA-relevant receptors (McInnes *et al.*, 2016; Mockridge *et al.*, 2017; Supplementary Table S3) which was subsequently integrated with expression data to create an RA-relevant diffusion model. Our ROC results found a remarkable AUC = 1 for receptor-TF pair PTPRZ1-NFKB1 (Fig. 4A), AUC = 0.94 each for PTPRZ1-JUN (Fig. 4B) and PTPRZ1-ETS2 (Fig. 4C) which accurately separates the TCZ treatment-resistant and responder patients (Fig. 4D–F). High AUC receptor-TF pairs are listed in Supplementary Data (Supplementary Table S5). GO analysis of the TFs identified in RA highlighted processes such as response to oxidative stress, cellular response to peptide, negative regulation of protein phosphorylation, etc. GO analysis highlights key immune processes associated with RA pathophysiology regulated by key TFs such as SPI1, RARA, PPARG, NFKB1, ETS1 and MAF (Supplementary Fig. S3; Giaginis *et al.*, 2009; Ikuta *et al.*, 2012; Kang *et al.*, 2017; Manuel Sánchez-Maldonado *et al.*, 2020; Zisakis et al., 2007).

## 4 Discussion

We have developed a diffusion model; a molecular pathway inspired method to model patient-specific treatment response. It creates a new feature space by using key TFs, receptors, biological prior information in the form of a PPI and gene expression data. This new feature space is a nonlinear transformation of the original gene expression variables, designed with the goal of being more relevant for describing cytokine signalling in UC. We have compared the ability of the new features to predict anti-TNF response in UC to two other methods. We used ‘nnet’, a machine learning method that has recently been used in biomarker discovery (Mallik *et al.*, 2020) and LIONESS, a regulatory network reconstruction method that estimates patient-specific regulatory connections. These methods represent two extremes in the analysis of biological data. The ‘nnet’ is a general machine learning method that controls overfitting by regularization model parameters. LIONESS–pandaR, however, uses a large amount of biological background information about TF-targets and PPI to estimate patient-specific GRNs. Although both the ‘nnet’ and the $t_{50}$ features may serve as useful biomarkers of drug response in independent datasets, the LIONESS-pandaR method fails to identify any biomarkers for drug response. This may be due to the large number of calculated network connection weights and the consequential penalty for multiple testing. Therefore, pandaR-LIONESS may lack the sensitivity to pick up the more subtle differences between subclasses of the patient samples, compared to the much larger difference between normal and inflamed tissue.

The diffusion-based features outperform the ‘nnet’ both in fitting to the training data and two out of three test datasets. This may be an indication that the combinations and transforms of the gene expression data derived from the signalling network topology might have more biological relevance than features obtained by a pure fit to the gene expression, despite the regularization penalties in ‘nnet’. Our method identified well-known pro-inflammatory receptors such as TNFRSF11B, OSMR, NRP1 and CCR2 which exhibited stronger connectivity (low $t_{50})$ to most inflammation-related TFs in UC patients with active inflammation than in non-UC controls and responders after treatment (Fig. 2A and Supplementary Fig. S4). These results must be interpreted with a caution as the responder samples may still contain residual microscopic inflammation and have lasting changes to their epithelial cells (Fenton *et al.*, 2021; Planell *et al.*, 2013). However, the goal of this analysis is to identify the receptor-TF pairs involved in active inflammation that requires treatment. Notably, our model also identified TFs ESRRA and HNF4A, which play an important role in the regulation of intestinal homeostasis. ESRRA is a regulator of intestinal homeostasis (Kim *et al.*, 2020), and HNF4A modulates inflammation in UC and maintains epithelial barrier integrity in the normal intestine (Ahn *et al.*, 2008; Barrett *et al.*, 2009).

Despite the good predictive performance, it is also noteworthy that both ‘nnet’ and the diffusion model performed worse on test dataset 3 (GSE73661). A dataset analysed with a different array design than the training dataset. This highlights the importance of robust and repeatable measuring processes for the practical use of complex gene expression-based biomarkers. Unfortunately, no large-scale modern RNA-seq datasets are currently available to test for predicting anti-TNF response in UC.

The diffusion model may also be susceptible to predictive errors because of the assumptions made in the initial data mining. We have chosen to focus on cytokines as the source of the inflammatory signal (Chen and Sundrud, 2016), but inflammatory diseases may also involve other signalling systems such as pattern recognition receptors and metabolic factors. The method is also highly simplified, ignoring molecular functions such as activation, repression and feedback loops, which are not considered explicitly. In addition, biological molecules of unknown function that may influence true network connectivity are ignored. Moreover, epigenetic factors have a crucial role in determining the transcriptional activation of genes targeted by a specific TF (Gibney and Nolan, 2010). However, obtaining epigenetic signatures for every individual patient is currently cost-prohibitive. Additionally, the evaluation does not take into account changes in the patient’s gene expression as the disease progresses. They may therefore be expected to give a more reliable prediction of short-term effects than in long-term remission. In conclusion, we assert that our diffusion model can be used to generate testable hypotheses applicable to UC and other autoimmune diseases such as RA, psoriasis and asthma. This framework outlines the receptor-TF-specific network connectivity which varies with the gene expression of each individual patient. Estimating the receptor-TF network connectivity associated with varied drug responses in disease subpopulations may yield valuable insights into a patient’s treatment outcome.

## Funding

This work has been supported by the Northern Norway Regional Health Authority [Strategisk-HN-10-16].

***Conflict of Interest*** none declared.

## Supplementary Material

vbab017_Supplementary_Data
