# Supplementary material for: Identifying anti-TNF response biomarkers in ulcerative colitis using a diffusion-based signalling model
Source: Bioinform Adv. 2021 Aug 18;1(1):vbab017. doi: 10.1093/bioadv/vbab017 (PMC9710619; doi:10.1093/bioadv/vbab017)
Supplement: vbab017_Supplementary_Data [file vbab017_supplementary_data.zip › Figure S1.pdf]

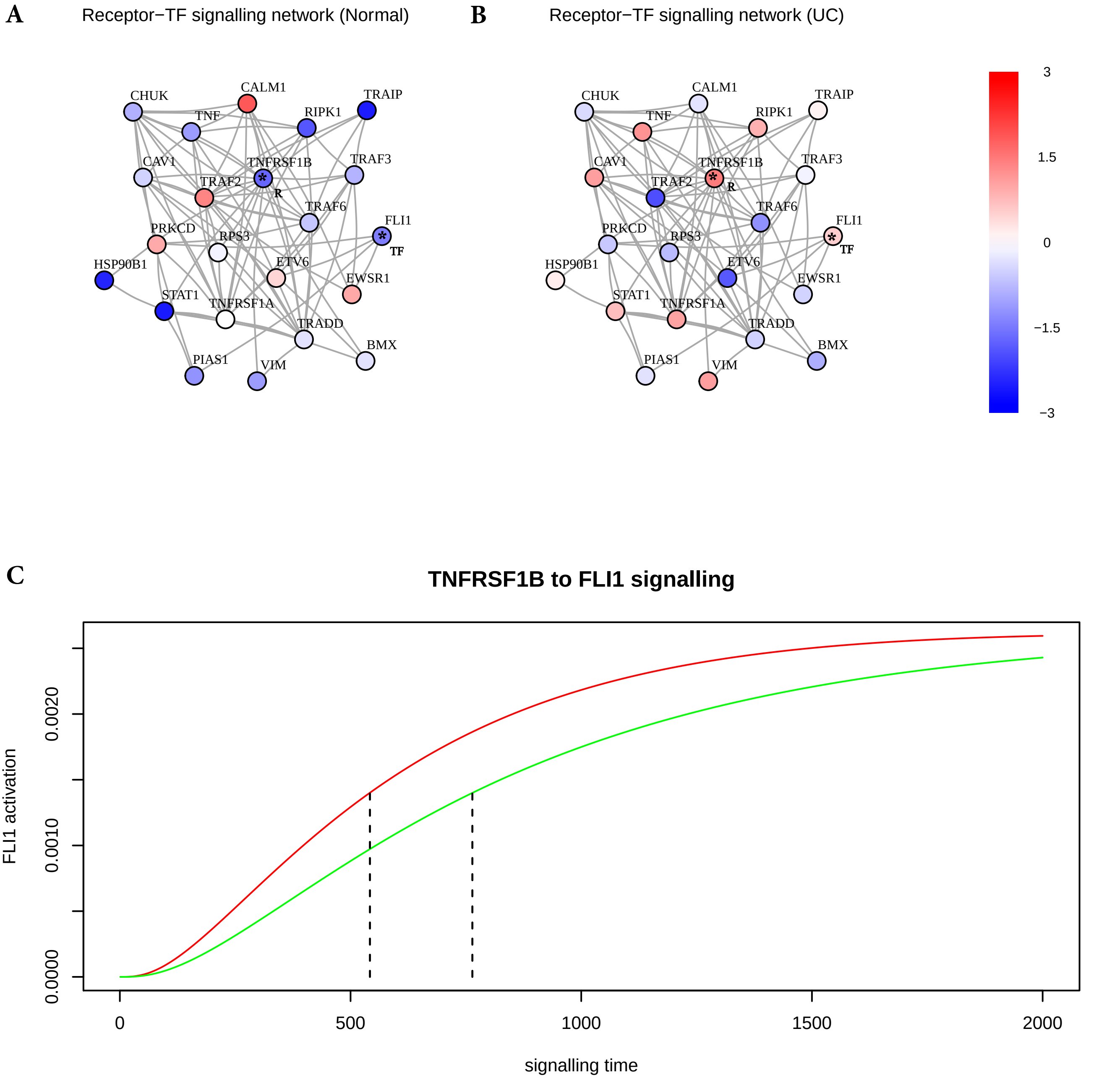

A
Receptor−TF signalling network (Normal)
B
Receptor−TF signalling network (UC)
CHUK
CALM1
TRAIP
TNF
RIPK1
CAV1
TNFRSF1B
TRAF3
TRAF2
R
TRAF6
FLI1
PRKCD
RPS3
TF
HSP90B1
ETV6
EWSR1
STAT1
TNFRSF1A
TRADD
BMX
PIAS1
VIM
3
1.5
0
−1.5
−3
C
TNFRSF1B to FLI1 signalling
FLI1 activation
0.0000
0.0010
0.0020
0
500
1000
1500
2000
signalling time
