# Supplementary material for: Identifying anti-TNF response biomarkers in ulcerative colitis using a diffusion-based signalling model
Source: Bioinform Adv. 2021 Aug 18;1(1):vbab017. doi: 10.1093/bioadv/vbab017 (PMC9710619; doi:10.1093/bioadv/vbab017)
Supplement: vbab017_Supplementary_Data [file vbab017_supplementary_data.zip › Supplementary_legends.docx]

## Supplementary Figure:

Figure S1. Comparison of ulcerative colitis (UC) and control sample based on signalling from the receptor TNFRSF1B to the TFs FLI1 (marked with *). (A) Signalling network with genes colour coded according to the average gene expression in UC sample and (B) normal sample. The node colour indicates the gene expression level. TF node with red colour represents high gene expression whereas blue represents low gene expression. (C) Line plot shows the amount of signal reaching the FLI1 TFs as a function of the number of time steps (t50) after a signal is induced on the receptor TNFRSF1B. Lines are colour coded according to the sample group. Dotted black vertical lines represent t50 for signal in UC patient and control.

*Figure S2. ROC analysis represents top AUC for receptor-TF pair that distinguish patients with before and after treatment group on training dataset.*

Figure S3. Gene Ontology Analysis (GO) analysis of TF-target genes relevant for Rheumatoid Arthritis (RA) treatment resistance. TF are selected based on the AUC score > 0.78. Dots represent key TFs and vertical right and left side illustrates TF-target gene enrichment terms.

Figure S4. Gene ontology analysis of TF-target genes relevant to anti-TNF therapy resistance. TFs are selected based on the AUC score > 0.90. Dots represent enrichment term with colour coding with blue shows higher significance, red shows lower significance enrichment of target genes of key TFs. The sizes of the dots represent the fraction of TF-target genes given each GO term. The X-axis shows a number of target genes per TF in immune category represented in the gene universe (shown in number).

Supplementary Table:

*Table S1. List of GWAS enriched IBD-relevant genes.*

Table S2 Datasets with anti-TNF response data for individual samples available.

Table S3. List of literature curated cytokines, receptors and pandaR identified Key TFs in UC (column no. 1-3) and RA (column no. 4-6).

Table S4. Annotation of identified key TFs in UC containing TF name, TF score, empirical P-value, and TF associated target genes respectively.

Table S5. Annotation of identified key TFs in RA containing TF name, TF score, empirical P-value, and TF associated target genes respectively.

Table S6. Receptor-TF pair for anti-TNF responder vs. non-responder groups obtained by diffusion model analysis followed by linear modelling (limma) containing t50, Average diffusion time, t – statistics, p-value, and adj. p-value respectively.

Table S7. Top AUC scores obtained by ROC analysis for receptor TFs pairs after diffusion model result in UC and RA with AUC > 0.84.

Table S8. Receptor-TF pair obtained by pandaR-LIONESS analysis followed by linear modelling (limma) in treatment responder. Vs. non-responder UC patients. Table header contains t50, Average diffusion time, t-statistics, p-value, and adj. p-value respectively.
