# Supplementary material for: Identifying anti-TNF response biomarkers in ulcerative colitis using a diffusion-based signalling model
Source: Bioinform Adv. 2021 Aug 18;1(1):vbab017. doi: 10.1093/bioadv/vbab017 (PMC9710619; doi:10.1093/bioadv/vbab017)
Supplement: vbab017_Supplementary_Data [file vbab017_supplementary_data.zip › Supplementary_methods.docx]

**Supplementary information:**

**Proposed method**

In this study, we propose quantifying patient specific network connectivities between pairs of genes to develop complex biomarkers that can predict treatment response. However, with more than 20,000 genes in the human genome the number of possible connections approaches 200 million. Therefore, it is necessary to identify a small number of biologically relevant connections to quantify, thus avoiding overfitting due to the large number of potential connections. Hence, we focus on the connectivities between disease relevant receptors and transcription factors (TFs) that regulate the expression of genes involved in the inflammatory process (**Figure 1A** and **Figure 1B**) using network diffusion. Network diffusion describes the gradual spread of an abstract signal throughout a network. Diffusion is a global network process that considers all available paths, not just direct links or shortest paths (Di Nanni *et al.*, 2020). Thus, the diffusion time represents the overall network connectivity from a receptor to a TF (**Figure 1A**).

**Material and Methods:**

The methods are briefly described ([see the supplementary method](https://github.com/Amy3100/netDiff1/tree/main/Supplementary) for details). Statistical analysis and processing of the data were performed using R version 3.6.3 ([www.r-project.org](http://www.r-project.org)). To identify relevant TFs, the Bioconductor R package, pandaR ([10.18129/B9.bioc.pandaR](https://doi.org/doi:10.18129/B9.bioc.pandaR) ) (Schlauch *et al.*, 2017) was used. IBD-relevant receptors were selected from the GWAS risk genes for IBD (**Table S1**). The comPPI database (Veres *et al.*, 2015) was used to create a signalling network connecting receptors to TFs. Diffusion analysis was performed on this network. The differential connectivity between sample groups was tested using linear modelling (Ritchie *et al.*, 2015). Gene ontology (GO) enrichment analysis was performed using the R package clusterProfiler.

**Data sources**

**Gene expression data**

The GEO was searched for datasets containing: Gene expression data from the colon biopsies obtained before treatment with anti-TNF and with treatment response data available. The detailed search protocol is available in supplementary methods. A search of the gene expression omnibus for gene expression data from ulcerative colitis patients was started by searching for UC gene expression datasets of human origin using the search string:

((Ulcerative Colitis)) AND "Homo sapiens"[porgn: txid9606])

This search identified 169 datasets potentially of interest. These were inspected manually to identify sets with available drug response data and gene expression from colonic biopsies from adult (non-paediatric) UC patients. Not blood or cells derived from blood samples.

The identified sets are listed in Table S2. Set GSE14580 is a subset of samples from GSE16879 and was therefore not used. GSE92415 was run on plated system with all controls run on a separate plate and was therefore not used due to problems in correcting for the plating bias in normalization.

**Transcription factor targets**

Regulatory motif binding information was obtained from the regulatory circuits database (Marbach *et al.*, 2016), which contains available TF binding sites in several tissues and cell types. The binding motif collection representing general immune cells (high level network “14_immune_organs.txt”) was chosen from a regulatory circuits database as a relevant representation of the inflammatory cells involved in UC.

**Protein-Protein Interaction database**

Protein-protein interaction (PPI) data were obtained from ComPPI database (Veres *et al.*, 2015). This is a cellular compartment-specific database of proteins and their interactions ([http://ComPPI.LinkGroup.hu](http://comppi.linkgroup.hu)). Only interactions with a confidence score > 0.6 were used in the network construction.

**Functional gene annotation**

Gene annotation was performed using the Bioconductor org.Hs.eg.db package version 3.12.0 [[10.18129/B9.bioc.org.Hs.eg.db](https://doi.org/doi:10.18129/B9.bioc.org.Hs.eg.db) ]. GO enrichment was performed using the clusterProfiler, Bioconductor package (Yu *et al.*, 2012).

**TF analysis using pandaR-LIONESS**

To identify key TFs, pandaR (Passing Attributes between Networks for Data Assimilation), was used. pandaR creates a gene regulatory network (GRN) with weighted edges between TFs and genes targets regulated by these TFs. To evaluate which TFs significantly contributed to the variation in gene expression, a null distribution regulation network edge weight was computed by randomizing the TF gene target information. Then, the resulting null distribution was used to calculate an empirical p-value for each TF. Sample-specific GRNs were created using LIONESS (Kuijjer *et al.*, 2019).

**Signalling network**

To model the signalling network connectivity between UC relevant cytokines and key transcription factors, a PPI network was constructed to connect key TFs to cytokine receptors that potentially initiate their activation. In the resulting signalling PPI network, the nodes represent the genes coding for the interacting proteins, and the edges represent physical interactions that may pass a biological signal. ComPPI was used to obtain protein-protein interactions. The network includes interactions involving the selected TFs (section 2.2), surface receptors which are known UC risk genes and signal transduction genes, such as kinases, that may contribute to passing information between the receptors and TFs.

**The diffusion model**

We chose to model the results of the biochemical events that occur during signal transduction using a network connecting cell surface receptors to TFs in the nucleus. The model is adapted from Fick’s law of chemical diffusion to a network structure. See e.g. (Jean Philibert, 2005) for a review. Consider a patient-specific signalling network with nodes representing proteins e.g., cytokines, receptors, and kinases create a signal transduction cascade. If a signal $S$, analogous to a concentration of a chemical in Fick’s law, is placed on a node $i$ the signal flux F along a network edge connecting node $i$ to node $j$ at a time $t$ is given by:

$${F(t)}_{i\to j} ={(S}_{i}-S_{j})\times E_{i}\times E_{j}$$

Where the edge connectivity weight, analogous to the diffusion constant in Fick’s law, is calculated using the patient’s normalized gene expression values, $E$, of the genes coding for the proteins $i$and$j$. The signal present at each protein node $i$connected to $J$other protein nodes $j \epsilon1..J$is then updated at time $t+1$using the sum of all fluxes:

$${S(t+1)}_{i}={S(t)}_{i} +\sum_{j = 1}^{J} {F(t)}_{i\to j}$$

The computation is initialized by setting all signal levels to zero and then placing one unit of signal on a starting receptor protein. The signal propagates through interconnected proteins throughout the network. To quantify the connectivity, we take the number of time steps to reach 50% of the maximum signal at the TF of the interest ${(t}_{50})$. This methodology was implemented in R (3.6.1) and ran to map connectivity between the selected receptors and the key TFs.

Table S2 Datasets with anti TNF response data for individual samples available

| Dataset | Drug | #Rsp | #Resist | Normals | Time [W] | Platform |
| --- | --- | --- | --- | --- | --- | --- |
| GSE16879 | Infliximab | 7 | 17 | 6 | 4-6 | Affymetrix Human Genome U133 Plus 2.0 Arrays |
| GSE14580 | Infliximab | 7 | 17 | 6 | 4-6 | Affymetrix Human Genome U133 Plus 2.0 Array |
| GSE12251 | Infliximab | 12 | 11 | 0 | 8 | Affymetrix Human Genome U133 Plus 2.0 Array |
| GSE23597 | Infliximab | 26 | 6 | 0 | 8 or 30 | Affymetrix Human Genome U133 Plus 2.0 Array |
| GSE92415 | Golimumab | 32 | 27 | 21 | 6 | Affymetrix HT HG-U133+ PM Array Plate |
| GSE73661 | Infliximab/Vedolizumab | 8 | 15 | 12 | 4-6 | Affymetrix Human Gene 1.0 ST Array |

## **REFERENCES**

Jean Philibert (2005) ‘One and a Half Century of Diffusion: Fick, Einstein, before and beyond’, *diffusiion-fundamentals*, (The Open-Access Journal for the Basic Principles of Diffusion Theory, Experiment and ApplicationDiffusion Fundamentals 2 (2005) 1.1-1.101).

Kuijjer, M. L. *et al.* (2019) ‘LionessR: Single sample network inference in R’, *BMC Cancer*, 19(1), pp. 4–9. doi: 10.1186/s12885-019-6235-7.

Marbach, D. *et al.* (2016) ‘Tissue-specific regulatory circuits reveal variable modular perturbations across complex diseases’, *Nature Methods*, 13(4), pp. 366–370. doi: 10.1038/nmeth.3799.

Di Nanni, N. *et al.* (2020) ‘Network Diffusion Promotes the Integrative Analysis of Multiple Omics’, *Frontiers in Genetics*, 11, p. 106. doi: 10.3389/fgene.2020.00106.

Ritchie, M. E. *et al.* (2015) ‘limma powers differential expression analyses for {RNA-sequencing} and microarray studies’, *Nucleic Acids Res.*, 43(7), p. e47.

Schlauch, D. *et al.* (2017) ‘Estimating gene regulatory networks with pandaR’, *Bioinformatics*, 33(14), pp. 2232–2234. doi: 10.1093/bioinformatics/btx139.

Veres, D. V. *et al.* (2015) ‘ComPPI: A cellular compartment-specific database for protein-protein interaction network analysis’, *Nucleic Acids Research*, 43(D1), pp. D485–D493. doi: 10.1093/nar/gku1007.

Yu, G. *et al.* (2012) ‘ClusterProfiler: An R package for comparing biological themes among gene clusters’, *OMICS A Journal of Integrative Biology*, 16(5), pp. 284–287. doi: 10.1089/omi.2011.0118.
