# Supplementary figures and images for: Identifying anti-TNF response biomarkers in ulcerative colitis using a diffusion-based signalling model
Source: Bioinform Adv. 2021 Aug 18;1(1):vbab017. doi: 10.1093/bioadv/vbab017 (PMC9710619; doi:10.1093/bioadv/vbab017)

ROC (TNFRSF11B\_ELF1): AUC = 0.91

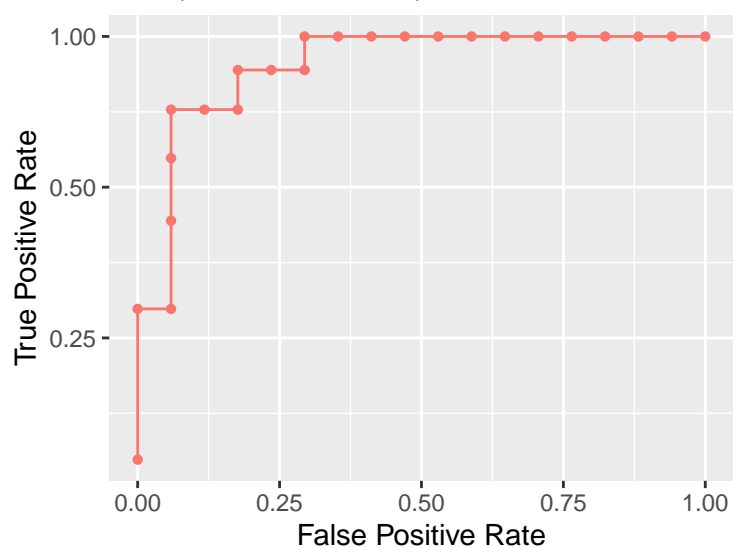

ROC (TNFRSF11B\_ZNF219): AUC = 0.91

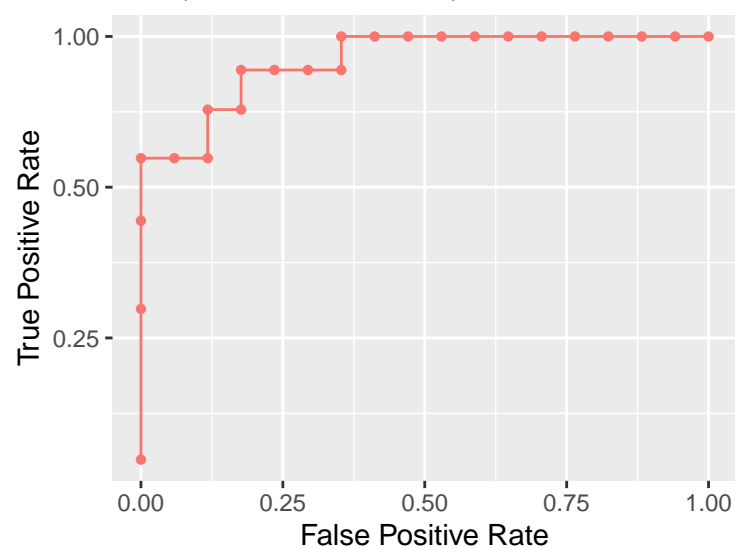

ROC (TNFRSF11B\_NFKB1): AUC = 0.91

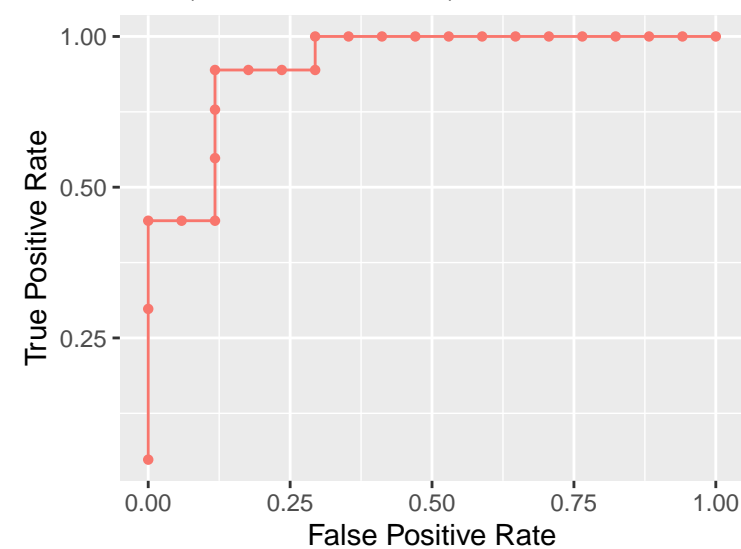

Supplement: vbab017_Supplementary_Data [file vbab017_supplementary_data.zip › Figure S2.pdf]

# Gene ontology of key TFs

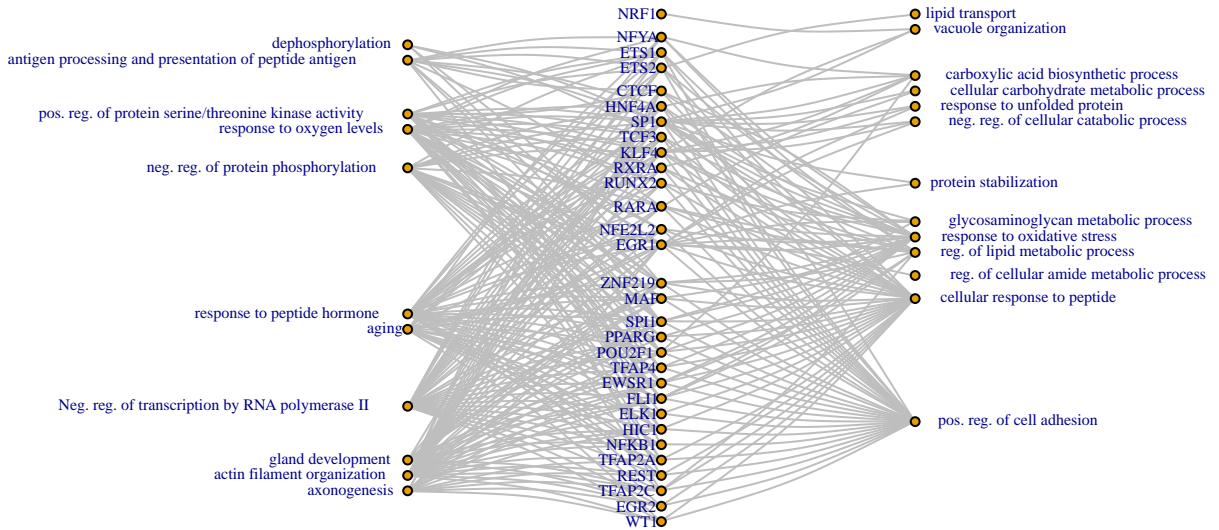

Supplement: vbab017_Supplementary_Data [file vbab017_supplementary_data.zip › Figure S3.pdf]

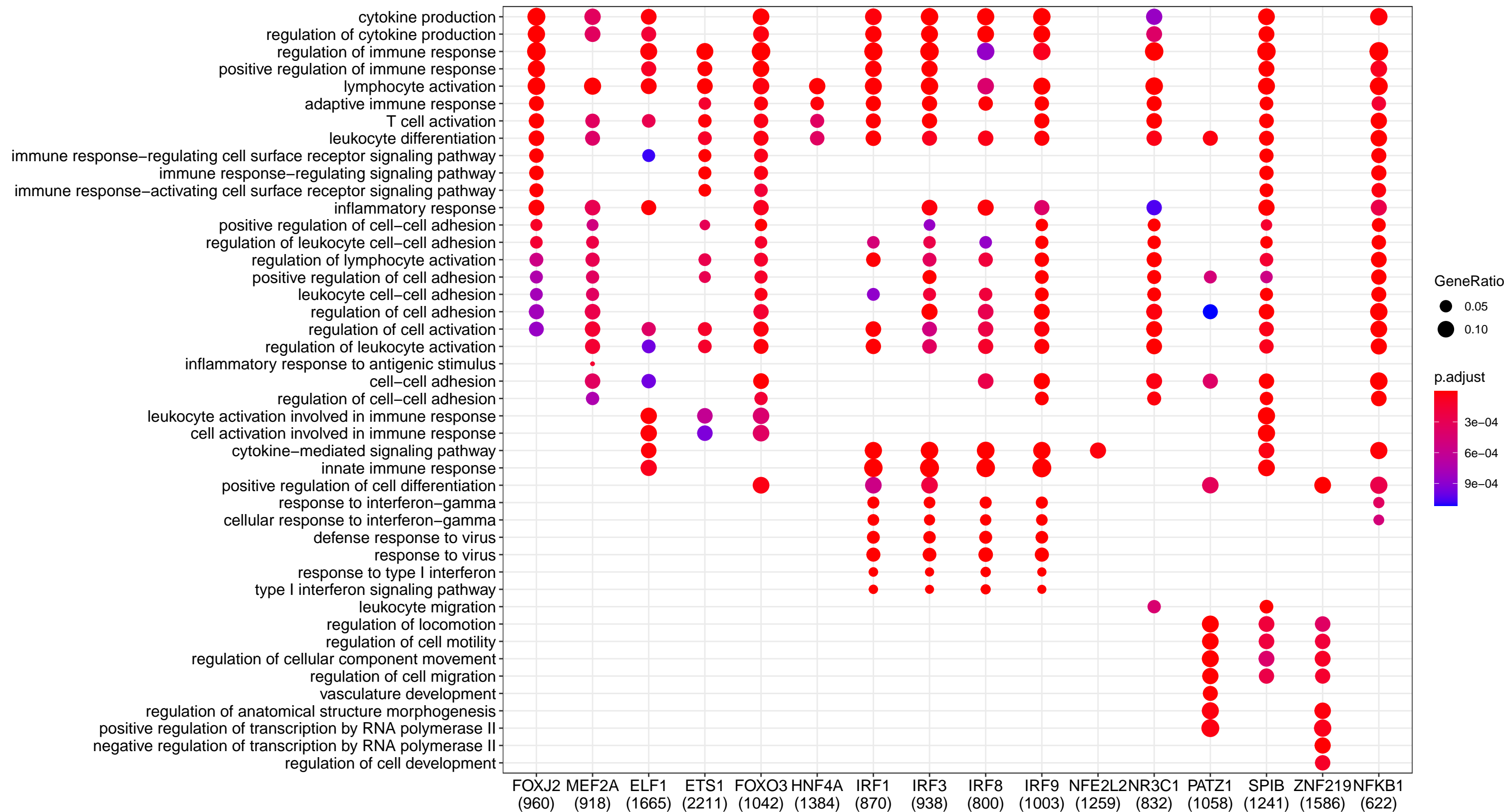

Supplement: vbab017_Supplementary_Data [file vbab017_supplementary_data.zip › Figure S4.pdf]
